# Effects of leisure activities and general health on the survival of older people: a cohort study in China

**DOI:** 10.3389/fpubh.2023.1273074

**Published:** 2023-10-03

**Authors:** Jianping Cai, Tingfa Hu, Lv Zhou, Hongye Jiang, Yumeng Gao

**Affiliations:** Department of Medical Insurance, Jinshan Hospital of Fudan University, Shanghai, China

**Keywords:** leisure activities, general health, survival, older people, mediation, moderation

## Abstract

**Objective:**

This study aimed to examine the influence of physical and cognitive leisure activities on the survival of older adults in China, while also exploring the potential mediating and moderating effects of general health.

**Methods:**

This study utilized the China Longitudinal Healthy Longevity Survey (CLHLS) datasets spanning from 2008 to 2018, and 10,347 eligible participants were included. The primary study outcome was all-cause mortality, and independent variables included physical leisure activities (PLA), cognitive leisure activities (CLA), and self-rated general health. Three sets of covariates were adjusted, including socio-demographic characteristics, health behaviors, and health status.

**Results:**

The longest survival time was the older people participating in PLA & CLA (mean = 50.31 months), while those participating in neither exhibited the lowest (mean = 29.60 months). Significant differences in survival status were observed in different types of leisure activities participation (Log-rank test, Chi-square = 576.80, *p* < 0.001). Cox regression indicated that PLA (HR = 0.705, 95% CI: 0.651–0.764), CLA (HR = 0.872, 95% CI: 0.816–0.933), and the both PLA & CLA (HR = 0.700, 95% CI: 0.656–0.747) were protective factors for the survival. Additionally, general health significantly moderated the relationship between PLA and reduced mortality risk (Coefficient = −0.089, *p* = 0.042). While CLA indirectly influenced the survival through general health (Coefficient = −0.023, *p* < 0.001). For the older people participating in PLA and CLA, general health played mediating (Coefficient = −0.031, *p* < 0.001) and moderating (Coefficient = −0.026, *p* = 0.013) role in the relationship between leisure activities and survival.

**Conclusion:**

Leisure activities and self-rated general health were important predictors of survival of the older adults, and general health exhibited a mediator and moderator in the relationship between leisure activities and survival status.

## 1. Introduction

Population aging has emerged as a pressing global social concern. China, in particular, is currently grappling with the largest aging population in the world. The latest census data from China in 2020 indicate that the proportion of those aged 65 years and above was 14%, thus solidifying China’s transition into a deeply aged society (1). This demographic shift presents many challenges to public health, caring for older adults, and societal burden, necessitating the provision of enhanced prevention, treatment, and rehabilitation services to address the health-related issues faced by older adults. Chronic diseases, cognitive decline, and difficulties in social integration are commonly encountered by older adults, which could lead to serious social problems (2, 3). Therefore, encouraging a suitable lifestyle for older adults, improving their health level, and achieving healthy aging are inevitable requirements to solve the above-mentioned challenges and problems.

The participation of older adults in leisure activities is an important component of a healthy aging society. Rocío (4) found that people who frequently engage in leisure time activities and/or leisure physical activities had a 19% lower mortality risk than those who rarely or never engage in those activities. A cohort study on older Japanese adults indicated that engagement in leisure activities showed a strong association with lowered mortality, with different types of leisure activities leading to various effects (5). Leisure activities encompass various categories, which can be classified into physical leisure activities (PLA) and cognitive leisure activities (CLA) based on the process and purpose of the activities (6, 7). However, the relationship between different types of leisure activities and the survival of older adults remains to be determined in China. Participation in PLA enhances muscle strength and maintains optimal physical functioning, whereas participation in CLA facilitates the establishment of social networks, reduces social isolation, and promotes emotional fulfillment among older adults (7, 8). Therefore, this study proposes the following research hypotheses:

*H1a*: Leisure activities positively enhance the survival of older adults.

*H1b*: Different types of leisure activities have various impact on improving the survival of older adults.

Self-rated general health is an essential aspect of health measurement in older people, as it provides valuable insights into their subjective understanding of their own overall well-being. It also serves as a comprehensive indicator that can reflect the health status across physiological, psychological, and social dimensions (9). A multi-center cohort study conducted in China, India, and Latin America revealed that older adults who reported poor self-rated general health exhibited a 142% higher risk of mortality within a 4-year follow-up period, as compared to those who reported moderate self-rated health (10). This is powerful evidence of the predictive value of self-rated general health in assessing mortality risk among older adults. Therefore, we propose the following hypothesis:

*H2*: Self-rated general health positively affects the survival of older adults.

Participating in leisure activities can have multiple beneficial effects on health promotion. However, there are few studies on the relationship between leisure activities and the general health of older adults in China. Older adults engaging in PLA can enhance their flexibility with physical activities and improve their immune system functions, which contribute to the prevention of chronic diseases such as cardiovascular disease, diabetes, and osteoporosis, thereby enhancing their physical health (11, 12). Additionally, CLA provide opportunities for older adults to pursue personal interests and goals. By participating in meaningful activities, older adults can experience a sense of achievement, satisfaction, and self-fulfillment, thereby enhancing their happiness and well-being (13). Thus, the following hypotheses can be inferred:

*H3*: Self-rated general health mediates the relationship between leisure activities and survival.

Due to variations in life experiences, physical fitness, and social contexts, older adults exhibit diverse health conditions. A favorable self-rated health status empowers older adults with the willingness, capability, and opportunities to engage in beneficial activities, thereby allowing leisure activities to promote their overall survival status to a greater extent. Conversely, a negative self-rated general health status may diminish the subjective motivation of older adults to participate in PLA and CLA, resulting in an increased risk of mortality (14, 15). From another perspective, the health status of older adults determines the strength of the relationship between leisure activities and survival. Therefore, we propose the following hypothesis:

*H4*: Self-rated overall health moderates the relationship between leisure activities and survival.

**Figure 1:**
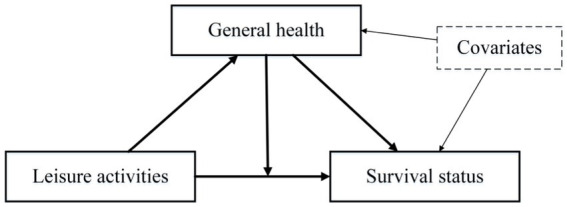
Potential study model based on hypotheses.

Using a 10-year cohort, this study aimed to examine the impact of leisure activities (PLA and CLA) and self-rated general health on the survival of older adults in China. Additionally, we explored the mediating and moderating effects of general health on the relationship between leisure activities and survival (Figure 1). The findings of this study are expected to offer valuable insights and recommendations for fostering the development of a healthy aging society in China in the future.

## 2. Methods

### 2.1. Study design and participants

The Chinese Longitudinal Healthy Longevity Survey (CLHLS) aimed to investigate the determinants of healthy longevity and to provide evidence for policy-making and intervention strategies to promote healthy aging in China. Initiated in 1998 by researchers from Peking University, the CLHLS adopted a longitudinal survey design with waves every 2–3 years. The CLHLS was a nationwide survey that covered half of the cities and counties in 23 provinces of China; this area had a total population of 1,156 million in 2010, which accounted for approximately 85% of the Chinese population. The CLHLS employed a multistage and stratified sampling strategy, which accurately reflected the broader population of older adults aged 65 and above in China. The survey collected data on demographic characteristics, health status, lifestyle factors, social support, family structure, economic conditions, and healthcare utilization (16).

This study utilized the CLHLS datasets spanning from the sixth wave (2008/2009, baseline) to the ninth wave (2017/2018). Varying age criteria exist among different countries, with some considering individuals aged 60 and above, and others opting for 65 and above. To enhance the generalizability of our study findings to a broader population, our focus was on individuals aged 65 years and older. There were 16,954 participants at baseline. After excluding 391 participants below the age of 65, 1761 with missing general health information, and 4,455 due to loss to follow-up, the final analysis included 10,347 eligible participants.

### 2.2. Variables and measurements

This study included one primary outcome variable, survival outcome; two independent variables, leisure activity and general health status; and 11 covariates, such as gender, age, and chronic diseases.

#### 2.2.1. Outcome definition

The primary outcome measure of this study focused on the occurrence of mortality across all causes. Mortality data were collected through follow-up surveys conducted in 2011, 2014, and 2018. In cases where death certificates were available, the date of death was verified using these documents. In instances where death certificates were unavailable, information provided by close family members was recorded. Subsequently, the duration of survival in months was calculated, representing the time interval between the date of the investigation and the participant’s recorded date of death.

#### 2.2.2. Independent variables

Leisure activities served as a crucial independent variable in this study. The CLHLS utilized six items to measure the participation of older adults in leisure activities: housework, gardening, pet care, reading newspapers/books, playing cards/mahjong, and watching television/listening to the radio (17). Among these, the first three items belonged to PLA, whereas the latter three belonged to CLA (18). Each item was assessed using a 5-point Likert scale. For example, the item “Do you do housework at present?” offered options ranging from “almost every day” to “never.” In both categories of leisure activities, if a participant responded “never” to a specific item, a value of 0 was assigned; otherwise, a value of 1 was assigned. Therefore, this study categorized the participation in leisure activities into four groups: no participation, only PLA, only CLA, and both PLA & CLA.

General health was another important independent variable in this study. The construct of general health encompassed three types of items: self-rated quality of life (b11), self-rated health (b12), and a question assessing changes in health since the previous year (b121) (19). Respondents were asked to rate their self-perceived quality of life and health as very good, good, fair, poor, or very poor, scored as 1–5 points, respectively. In data processing, responses falling under “cannot answer” were treated as missing data. The item measuring health change (b121) offered five response options: much better, slightly better, almost the same, slightly worse, and much worse, scored as 1–5 points, respectively. Next, the negative health items were positively transformed by reversing their scores. The scores of the three items were then summed, creating a score range of 3 to 15, with higher scores indicating better general health.

#### 2.2.3. Covariates

This study encompassed three sets of potentially confounding variables: socio-demographic characteristics, health behaviors, and health status. Socio-demographic characteristics comprised gender, age, education, marital status, and household income. Education involved gathering data on the number of years of formal education completed by the participants. Individuals with no formal education were classified as illiterate. Marital status was classified as married, unmarried, or widowed. Household income was categorized into three levels based on self-reported annual income: <10,000, 10,000–30,000, and > 30,000 yuan. Health behaviors included smoking (yes/no), alcohol consumption (yes/no), and regular physical exercise (yes/no). Health status encompassed body mass index (BMI), occurrence of severe illness in the past two years, and numbers of chronic diseases. BMI was calculated using the formula [weight (in kilograms)/height (in meters)^2]. The presence of several chronic diseases was assessed by asking the respondents, “Do you suffer from the following diseases?” Participants could choose from 22 options, such as hypertension, diabetes, and stroke.

### 2.3. Statistical analysis

The variables with a normal distribution were described using the mean ± standard deviation (SD) within each leisure activity type. Categorical variables were presented as frequencies and percentages (%). For each type of leisure activity, we calculated the means and their corresponding 95% confidence intervals (CIs) to determine the survival time.

Furthermore, to illustrate the survival trends over time, Kaplan–Meier survival analyses were conducted. The equality of survival time among older adults with different types of leisure activities was examined using the log-rank test. Additionally, after adjusting for socio-demographic characteristics, health behaviors, and health status, a Cox regression analysis was performed to explore the risk factors associated with mortality in older adults. The results were reported in terms of hazard ratio (HR) along with its standard error (SE). The statistical assumption of the Cox regression was assessed and found to be supported.

In this study, the relationship between leisure activities and survival was examined while considering the mediating and moderating effects of general health. The *med4way* command in Stata software was used to calculate these effects after adjusting for covariates (20). This command is suitable for various types of outcomes and mediators. In this case, survival data were the outcome variable, estimated using Cox regression, and linear regression was used for the mediator, general health. The *med4way* command can decompose the overall effect into four components: no mediation or interaction, only interaction, both mediation and interaction, and only mediation. The focus of this study was reporting the mediation and interaction effects. More information about the *med4way* command can be found elsewhere (20).

All the data analysis was performed using Stata 14.0 MP version (Stata Corp LP, College Station, TX, United States). All tests were two-sided, and a *value of p* of <0.05 was considered statistically significant.

## 3. Results

### 3.1. Characteristics of the older people at baseline

In this study, there were 4,644 male and 5,703 female older adults. The mean age was 86.67 years (SD = 11.02). A total of 6,479 participants were illiterate, and 7,098 were unmarried. More than half of the older adult population had an annual income below 10,000 yuan. The majority of the individuals were non-smokers (*N* = 8,450) and non-drinkers (*N* = 8,448), but 7,567 did not engage in regular physical exercise. The average score for general health was 8.27 (SD = 1.97), and the average BMI score was 20.22 (SD = 3.53). Among the participants, 1,664 had experienced a significant illness in the past two years. A total of 4,600 individuals had no chronic diseases, whereas 3,267 had one chronic disease, and 2,480 had two or more chronic diseases simultaneously. Additionally, the characteristics above with various leisure activities were calculated in four groups, with detailed information shown in Table 1.

**Table 1:** Characteristics of the older people in different leisure activities at baseline.

Variables	Dimensions	Total, N / Mean ± SD	Type of leisure activities, N(%)/Mean ± SD			
			None	Only PLA	Only CLA	PLA & CLA
Sex	Male	4.644	563(12.12)	356(7.67)	1,182(25.45)	2,543(54.76)
	Female	5.703	1.284(22.51)	833(14.61)	992(17.39)	2.594(45.48)
Age	–	86.67 ± 11.02	90.98 ± 7.78	88.62 ± 9.22	90.94 ± 9.19	81.43 ± 10.32
Illiteracy	Yes	6.479	1.495(23.07)	964(14.88)	1.270(19.60)	2.750(42.44)
	No	3.868	352(9.10)	225(5.82)	904(23.37)	2.387(61.71)
Marriage status	Married	3.249	206(6.34)	249(7.66)	537(16.53)	2.257(69.47)
	Unmarried	7.098	1.641(23.12)	940(13.24)	1.637(23.06)	2.880(40.57)
Income (yuan/year)	<10,000	5.422	1.040(19.18)	831(15.33)	894(16.49)	2.657(49.00)
	10,001 ~ 30,000	3.092	512(16.56)	201(6.50)	766(24.77)	1.613(52.17)
	>30,000	1833	295(16.09)	157(8.57)	514(28.04)	867(47.30)
Smoking	Yes	1897	176(9.28)	155(8.17)	431(22.72)	1.135(59.83)
	No	8.450	1.671(19.78)	1.034(12.24)	1743(20.63)	4.002(47.36)
Drinking	Yes	1899	193(10.16)	153(8.06)	432(22.75)	1.121(59.03)
	No	8.448	1.654(19.58)	1.036(12.26)	1742(20.62)	4.016(47.54)
Physical activities	Yes	2.780	200(7.19)	217(7.81)	586(21.08)	1777(63.92)
	No	7.567	1.647(21.77)	972(12.85)	1.588(20.99)	3.360(44.40)
General health	–	8.27 ± 1.97	8.18 ± 1.89	8.01 ± 1.88	8.74 ± 2.15	8.82 ± 1.96
BMI	–	20.22 ± 3.53	19.17 ± 3.45	19.32 ± 3.19	20.22 ± 3.45	20.80 ± 3.54
Serious illness in past 2 years	No	8.683	1.523(17.54)	1.026(11.82)	1750(20.15)	4.384(50.49)
	Yes	1.664	324(19.47)	163(9.80)	424(25.48)	753(45.25)
Chronic diseases	No	4.600	886(19.26)	600(13.04)	857(18.63)	2.257(49.07)
	One	3.267	542(16.59)	356(10.90)	716(21.92)	1.653(50.60)
	Two or more	2.480	419(16.90)	233(9.40)	601(24.23)	1.227(49.48)

SD, standard deviation; PLA, hysical leisure activities; CLA, cognitive leisure activities. BMI, body mass index.

### 3.2. Survival time of the older people participating in different leisure activities

The average survival duration of individuals who did not engage in any leisure activities was 29.60 months (SE = 0.61). For individuals who only participated in PLA, the average survival duration was 44.48 months (SE = 0.97), which was higher than the average survival duration of 36.99 months (SE = 0.62) for those who only engaged in CLA. In contrast, individuals who simultaneously participated in both PLA & CLA had an average survival duration of 50.31 months (SE = 0.57). Overall, the average survival duration for individuals was 41.68 months, with a 95% CI ranging from 41.00 to 42.35 (Table 2).

**Table 2:** Survival months of the older people in different leisure activities.

Groups	Mean*	S.E.	95% CI	
			Low	High
No leisure activities	29.60	0.61	28.40	30.80
Only PLA	44.48	0.97	42.58	46.39
Only CLA	36.99	0.62	35.78	38.21
PLA & CLA	50.31	0.57	49.19	51.44
Total	41.68	0.35	41.00	42.35

*Largest observed analysis time is censored, mean is underestimated. PLA, physical leisure activities; CLA, cognitive leisure activities; S.E., standard error; CI, confidence interval.

### 3.3. Kaplan–Meier survival estimates of the older people

**Figure 2:**
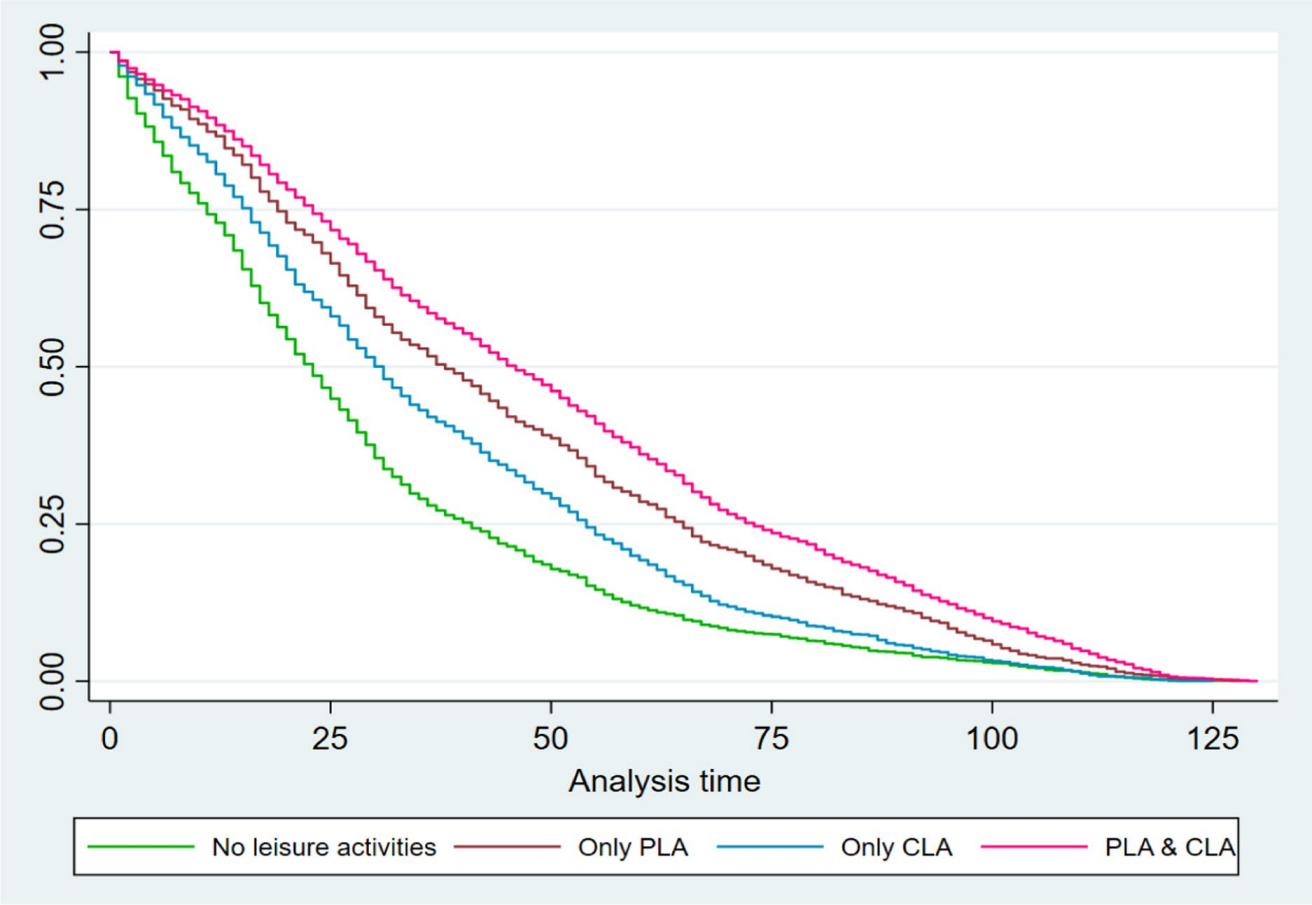
Kaplan–Meier survival estimates of the older people participating in different type of leisure activities.

Kaplan–Meier survival analysis was employed to estimate the survival status of individuals participating in different types of leisure activities. Overall, significant differences in survival status were observed among individuals participating in different types of leisure activities (log-rank test, chi-square = 576.80, *p* < 0.001). Further analysis using the log-rank test revealed variations in survival rates between different leisure activity groups. Individuals simultaneously participating in both PLA & CLA exhibited the highest survival rates (log-rank test, chi-square = 25.53, *p* < 0.001), followed by those engaging in only PLA (log-rank test, chi-square = 42.73, *p* < 0.001), and then by those involved in only CLA (log-rank test, chi-square = 55.65, *p* < 0.001). The poorest survival status was observed in individuals who did not participate in either type of leisure activity (Figure 2).

### 3.4. Cox regression for determinants of survival status

The Cox regression results indicated that compared to individuals refusing leisure activities, participation in PLA (HR = 0.705, 95% CI: 0.651–0.764), CLA (HR = 0.872, 95% CI: 0.816–0.933), and both PLA & CLA (HR = 0.700, 95% CI: 0.656–0.747) were protective factors for the survival of individuals. Older adults with better general health also had a lower risk of mortality (HR = 0.965, 95% CI: 0.953–0.967). Female individuals had a lower risk of death compared to males (HR = 0.855, 95% CI: 0.808–0.905). However, this study showed that individuals who engaged in frequent physical exercise had a higher risk of mortality (HR = 1.111, 95% CI: 1.053–1.172). Statistical significance was observed in mortality risk for older people who experienced a serious illness in the past two years (HR = 1.071, 95% CI: 1.006–1.140). No statistical significance (*p* > 0.05) was observed for other variables in relation to the survival status of older people. Therefore, the hypotheses H1a, H1b and H2 were supported (Table 3).

**Table 3:** Cox regression for determinants of survival status in the older people.

Variables	HR	S.E.	*Z*	*p*	95% CI	
					Low	High
*Leisure activities (Ref: No leisure activities)*						
PLA	0.705	0.029	−8.60	<0.001	0.651	0.764
CLA	0.872	0.030	−3.98	<0.001	0.816	0.933
PLA&CLA	0.700	0.023	−10.75	<0.001	0.656	0.747
General health	0.965	0.006	−5.89	<0.001	0.953	0.976
Female	0.855	0.025	−5.38	<0.001	0.808	0.905
Illiteracy	0.977	0.028	−0.81	0.418	0.924	1.033
Married	1.048	0.032	1.53	0.125	0.987	1.113
*Income (Ref: <10,000 yuan/year)*						
10,001 ~ 30,000	1.003	0.027	0.12	0.904	0.953	1.057
>30,000	1.026	0.032	0.82	0.414	0.965	1.091
Smoking	1.039	0.034	1.16	0.244	0.974	1.109
Drinking	1.015	0.032	0.46	0.645	0.953	1.081
Physical activities	1.111	0.030	3.87	<0.001	1.053	1.172
*BMI (Ref: Normal 18.5 ~ 23.9)*						
Under-weight	0.974	0.024	−1.07	0.286	0.928	1.022
Over-weight	1.017	0.040	0.43	0.669	0.941	1.098
Serious illness in past 2 years	1.071	0.034	2.13	0.033	1.006	1.140
*Number of Chronic diseases (Ref: No chronic diseases)*						
One	1.000	0.027	0.00	0.998	0.949	1.054
Two or more	0.996	0.031	−0.14	0.887	0.937	1.058

PLA, physical leisure activities; CLA, cognitive leisure activities; S.E., standard error; CI, confidence interval. BMI, body mass index; HR, hazard ratio.

### 3.5. Mediation and moderation role of general health in mortality

Using the *med4way* command in Stata software, we explored the moderating and mediating effects of general health. Firstly, in comparison to individuals who did not engage in any leisure activities, participation in PLA significantly reduced the risk of death directly (Coefficient = 0.192, *p* < 0.001). Furthermore, general health significantly moderated the relationship between PLA and reduced mortality risk (Coefficient = −0.089, *p* = 0.042). Secondly, relative to individuals who did not participate in any leisure activities, engagement in CLA directly and significantly lowered the risk of mortality (Coefficient = −0.115, *p* < 0.001). Moreover, CLA indirectly influenced the risk of mortality through general health, which was also significant (Coefficient = −0.023, *p* < 0.001). Lastly, simultaneous participation in both PLA & CLA significantly reduced the risk of mortality, as compared to no participation in leisure activities (Coefficient = −0.291, *p* < 0.001). This effect was mediated through general health, leading to a significant impact on the risk of mortality (Coefficient = −0.031, *p* < 0.001). Additionally, it was observed that general health served as a moderating variable in the relationship between leisure activities and survival status (Coefficient = −0.026, *p* = 0.013).

Therefore, the hypotheses H3 and H4 were supported (Table 4).

**Table 4:** Mediation and moderation role of general health on mortality status.

Specific effects	Coefficient	S.E.	*Z*	*p*	95% CI	
					Low	High
*Effect of PLA on the survival*						
PLA → Mortality(direct effect)	−0.192	0.051	−3.800	<0.001	−0.292	−0.093
PLA → GH → Mortality	0.001	0.004	0.310	0.754	−0.006	0.009
PLA × GH → Mortality	−0.089	0.044	−2.040	0.042	−0.174	−0.003
*Effect of CLA on the survival*						
CLA → Mortality(direct effect)	−0.115	0.031	−3.690	<0.001	−0.177	−0.054
CLA → GH → Mortality	−0.023	0.006	−3.720	<0.001	−0.036	−0.011
CLA × GH → Mortality	−0.003	0.014	−0.220	0.825	−0.029	0.024
*Effect PLA&CLA on the survival*						
PLA&CLA → Mortality(direct effect)	−0.291	0.025	−11.730	<0.001	−0.339	−0.242
PLA&CLA → GH → Mortality	−0.031	0.008	−4.040	<0.001	−0.046	−0.016
PLA&CLA × GH → Mortality	−0.026	0.010	−2.500	0.013	−0.047	−0.006

PLA, physical leisure activities; CLA, cognitive leisure activities; S.E., standard error; CI, confidence interval; GM = mental health. “×“meant the moderating effects, and → meant the regression path.

## 4. Discussion

In the social and cultural context of China, individuals reaching retirement age are confronted with the challenge of selecting suitable lifestyles. Among the available options, engaging in leisure activities plays a significant role in the lives of many older people and exerts a notable influence on their health and survival outcomes. However, few research exists in China concerning the relationship between leisure activity types, health status, and survival among older adults. This knowledge gap hinders the provision of guidance and recommendations regarding leisure choices in later life. To address this gap, the present study used data from the CLHLS, spanning a 10-year duration, with the primary objective of examining the impact of diverse leisure activity participation on mortality rates among older adults. Moreover, this study first explored the moderating and mediating effects of self-rated general health in the association between leisure activities and survival status. By drawing on the study findings, practical implications can be derived to assist older individuals in making decisions regarding suitable leisure activities, thereby offering valuable evidence to enhance both their navigation through later life and their overall well-being.

Our study participants exhibited diverse survival status based on their engagement in various leisure activities. On the one hand, the findings of this study revealed that participating in leisure activities was associated with an average survival time that was approximately 20 months longer than that of non-participants. This difference could be because engaging in leisure activities is linked to a favorable lifestyle, improved mental health, and a reduced risk of chronic diseases (6, 21). On the other hand, significant differences in survival status were observed among older adults involved in different types of leisure activities. The results indicated that the highest survival rate was among those participating in both PLA & CLA, followed by individuals engaged in PLA only, whereas those solely participating in CLA exhibited the lowest survival rate. Encouraging greater participation in leisure activities among older adults is a pivotal approach for promoting healthy aging (17). However, China still faces inadequate investment in infrastructure for facilitating older adult engagement in leisure activities, which undermines the motivation of older adults to actively engage in diverse leisure pursuits alongside a lack of feasible and effective intervention measures (22–24). Therefore, it is imperative to develop public health policies that foster the development of leisure activities for older adults, with a focus on expanding accessibility and coverage to ensure broader participation.

Our survival analysis suggested that self-rated general health was an important predictive factor of mortality in older adults. By assessing their own general health status, older adults can provide valuable insights into their overall well-being and health conditions (25, 26). Moreover, self-rated general health serves as a comprehensive measure that encompasses physical, mental, and social dimensions of health, thereby capturing the multidimensional aspects that influence mortality outcomes in this population (27, 28). Consequently, efforts should be directed toward promoting and maintaining positive self-rated general health among older adults, as this can contribute to reducing mortality rates and enhancing the overall quality of life in this population. Additionally, male sex, engaging in too much physical exercise, and a serious illness in the past two years were proven to be negatively associated with survival in the older adults, which was in line with previous studies (29, 30). Therefore, more public attention should be paid to these populations to prevent early death.

Furthermore, we explored the moderating and mediating effects of general health. Compared to older adults who did not engage in any leisure activities, participation in only PLA exhibited an interactive effect on general health, resulting in a reduced mortality risk. Older adults with better health conditions had a greater capacity to engage in multiple PLA, which contributed to extending their lifespan. However, our study found no significant impact of PLA on the health of older adults. Housework, which is a component of PLA, has been associated with increased exposure to potentially harmful substances such as kitchen fumes and inhalable dust, as highlighted by previous research (31, 32). This factor could potentially explain the lack of observed mediating effects in our results. In contrast, CLA significantly improved the overall health of older individuals and subsequently increased their survival rates. As older adults reduce their participation in physical labor, factors influencing health primarily stem from the psychological domain (33, 34). Engaging in CLA significantly enhanced the mental health of older individuals, bolstered their overall self-assessment of health, and consequently contributed to improved survival outcomes.

Finally, this study found that, compared to the older adult population who do not participate in any leisure activities, general health can serve as both a mediator and moderator of the impact of two types of leisure activities on survival status. On the one hand, participating in both types of leisure activities can help to comprehensively improve the physiological, psychological, and social adaptation dimensions of the health status of older adults, improve their quality of life, and reduce risk factors for death (35, 36). On the other hand, good health can help strengthen the relationship between leisure activities and survival rate. In other words, health status plays an important role as a catalyst between multidimensional leisure activities and longevity (37). Therefore, we should strive to create basic conditions conducive to the multidimensional participation of older adults in leisure activities, establish a monitoring and evaluation system for their health status, and take comprehensive interventions to improve their survival rate and promote healthy aging.

However, some limitations of this study should be mentioned. First, the measures of PLA and CLA are not exhaustive. Due to constraints in the scope of CLHLS data collection, other measures of leisure activities were not incorporated into the study, potentially introducing bias into the estimates. Second, over a quarter of the cohort was lost to follow-up, primarily due to urban development and relocation, which also may have introduced bias into our results. Third, the outcome examined in this study was all-cause mortality, and further research is needed to estimate the specific mortality risks associated with leisure activities. Finally, accounting for a broader set of confounders could have strengthened our results. Unmeasured (e.g., medical treatment and diet) or unknown potential covariates may have confounded the relationship between leisure activities, general health, and mortality.

## 5. Conclusion

All our hypotheses were verified by empirical evidence. Specifically, the findings revealed that older adults who engaged in both PLA and CLA exhibited the highest survival rates, followed by those involved in PLA alone, whereas individuals solely participating in CLA demonstrated the lowest survival rates. A survival analysis further showed the significance of self-rated general health and leisure activities as critical predictive factors influencing the survival of older adults. Importantly, this study reveals the dual role of general health as both mediator and moderator in the relationship between leisure activities and survival status. The implications of these findings extend to various stakeholders, including policymakers, healthcare providers, and researchers, who can leverage these insights to inform evidence-based interventions and strategies aimed at promoting healthy aging and enhancing the longevity and well-being of the older population in China.

## Data availability statement

Publicly available datasets were analyzed in this study. This data can be found at: http://opendata.pku.edu.cn/dataverse/CHADS&lt.

## Ethics statement

The studies involving humans were approved by Duke University Health System Institutional Review Board. The studies were conducted in accordance with the local legislation and institutional requirements. The participants provided their written informed consent to participate in this study.

## Author contributions

JC: Writing – original draft, Formal analysis, Software, Visualization. TH: Resources, Writing – review & editing. YG: Writing – original draft, Conceptualization, Data curation, Project administration, Supervision, Validation. HJ: Funding acquisition, Software, Writing – review & editing. LZ: Funding acquisition, Software, Writing – review & editing.
